# The Prevalence and Comorbidities of Self-Reported Mental Health Disorders Among Primary Healthcare Attendees in Riyadh, Saudi Arabia: A Cross-Sectional Survey

**DOI:** 10.3390/healthcare14060817

**Published:** 2026-03-23

**Authors:** Noof Alwatban, Mamdouh M. Shubair, Mariam A. A. Elmetwally, Bashar Mahmoud, Badr F. Al-Khateeb, Khadijah Angawi, Abeer Almudaihim, Nada Kareem Alenazi, Sahar Fahad Binhowaimel, Ashraf El-Metwally

**Affiliations:** 1 College of Public Health & Health Informatics, King Saud bin Abdulaziz University for Health Sciences, Riyadh 14611, Saudi Arabia; watbann@ksau-hs.edu.sa; 2 Ministry of the National Guard-Health Affairs, Riyadh 11426, Saudi Arabia; khateebb@mngha.med.sa (B.F.A.-K.);; 3 King Abdullah International Medical Research Center, Riyadh 11481, Saudi Arabia; 4 School of Health Sciences, University of Northern British Columbia (UNBC), 3333 University Way, Prince George, BC V2N 4Z9, Canada; mamdouh.shubair@unbc.ca; 5 Department of Psychology and Global One Health Academy, North Carolina State University, Raleigh, NC 27606, USA; mariam.elmetwally@gmail.com; 6 College of Medicine, Ibn Sina University for Medical Sciences, Amman 16197, Jordan; bashar.qn.mahmoud@outlook.com; 7 College of Medicine, King Saud bin Abdulaziz University for Health Sciences, Riyadh 14611, Saudi Arabia; 8 Department of Health Services and Hospital Administration, Faculty of Economics and Administration, King Abdulaziz University, Jeddah 21589, Saudi Arabia; kkangawi@kau.edu.sa; 9 College of Applied health Sciences, King Saud bin Abdulaziz University for Health Science, Riyadh 14611, Saudi Arabia; 10 Ministry of Health, Riyadh Second Health Cluster, Riyadh 12231, Saudi Arabia; nada.al.enazi11@gmail.com (N.K.A.); saharbinhowaimel@gmail.com (S.F.B.)

**Keywords:** mental disorders prevalence, sociodemographic factors, comorbidities, primary health care, Saudi Arabia

## Abstract

**Background/Objectives**: This study aimed to determine the prevalence of self-reported prior diagnosis of mental health disorders and identify their sociodemographic, behavioral, and comorbidity-related determinants among primary healthcare attendees in Riyadh, Saudi Arabia. **Methods**: A cross-sectional survey was conducted among 14,239 Saudi residents visiting primary healthcare centers in Riyadh. Univariate analysis (*p*-value < 0.25) and multivariable logistic regression (*p*-value < 0.05) were employed to identify independent predictors of self-reported prior diagnosis of mental health disorders. **Results**: The prevalence of self-reported prior diagnosis of mental health disorders was 2.5% (95% CI: 2.24–2.76%). Multivariable analysis revealed several significant independent predictors. Individuals aged less than 50 years (AOR = 1.47, 95% CI: 1.19–1.83, *p* < 0.001) and females (AOR = 1.98, 95% CI: 1.56–2.50, *p* < 0.001) had significantly higher odds of reporting a prior diagnosis of a mental health disorder. The presence of health insurance was also found to be associated with increased odds (AOR = 1.85, 95% CI: 1.48–2.30, *p* < 0.001). Most importantly, smoking (AOR = 4.45, 95% CI: 3.22–6.15, *p* <0.001), hypertension (AOR = 2.32, 95% CI: 1.61–3.34, *p* <0.001), obesity (AOR = 9.40, 95% CI: 6.96–12.70, *p* <0.001), hypercholesterolemia (AOR = 2.84, 95% CI: 1.98–4.07, *p* < 0.001) and heart disease (AOR = 12.74, 95% CI: 9.25–17.56, *p* < 0.001) all were strong independent predictors of a prior diagnosis of a mental health disorder. **Conclusions**: The study identifies a measurable burden of self-reported prior mental health diagnoses among primary healthcare attendees in Riyadh, with higher odds observed among younger individuals, women, smokers, and those with chronic physical conditions. These findings highlight the importance of integrating mental health screening and management into primary healthcare services in Saudi Arabia.

## 1. Introduction

Mental disorders represents a profound global public health risk impacting almost 970 million individuals globally in 2019, and the most common conditions include anxiety and depression [1,2]. These are not just isolated illnesses, but the most important contributors to the global burden of disease, where one of every six lived with a disability that results in a shorter life expectancy of 10 to 20 years among persons with severe mental health conditions [1]. It does not just affect individual health, but also has ripple effects on family relationships, socialization, and productive work environments [1]. More worryingly, there is still a significant treatment gap as more than 75% of patients in the low- and middle-income countries do not access the necessary mental healthcare [2].

Over the period of 1990 to 2019, mental disorders have had a significant burden in the Middle East and North Africa (MENA) region [3]. Extensive research has consistently shown that comorbid mental health problems are extremely common, and they are linked with worse symptom severity, reduced social support, reduced quality of life, and a heightened risk of suicidal thoughts and suicide [4,5,6]. This growing trend highlights the urgency to provide thorough epidemiological data in order to inform effective public health strategies. It is important to understand these complex interactions to provide holistic care to the patient and allocate resources.

In the Kingdom of Saudi Arabia (KSA), mental disorders are a significant health issue in the population, with the prevalence of depression being the most prevalent mental health issue [7]. Estimates of national surveys show that a large segment of the Saudi population, approximately more than one-third, will have a mental health condition at some point in their lives [8,9,10]. This high prevalence has gone hand in hand with high unmet mental health service needs [11,12]. Cultural influences such as the belief that mental illness can be attributed to non-medical causes, such as spiritual forces, and related stigmas, and the inclination to seek help through religious rather than professional means, can also play a role in low-help-seeking behaviors and attitudinal barriers to treatment [13,14,15].

Primary healthcare (PHC) centers are the foundation of the healthcare system of any country, as they are often the initial point of contact of individuals seeking medical services [16]. PHC providers play a crucial role in early detection, early intervention, and long-term management of mental health issues because a considerable percentage of mental illnesses are first identified and treated in these environments [16]. Nevertheless, the current literature related to mental disorders in Saudi Arabia has mainly concentrated on a specific population (e.g., hospital patients, students) or specific disorders and, in many cases, it does not provide a complete national community-based data [17,18,19]. Although there are some studies which have covered barriers to care and the incorporation of mental health into PHC, a clear visualization of the present prevalence and trends of comorbidity among those with access to primary healthcare has yet to take shape.

In this study, the researchers attempt to fill these vital gaps in the form of a cross-sectional survey of patients who visit primary healthcare centers within the city of Riyadh. It aims at capturing a representative sample that is already in contact with the healthcare system by targeting those who present to PHC settings. The study will present current prevalence rates of the different mental disorders, but, more importantly, it will shed light on the comorbidity levels and types of these diseases in this particular setting. The results will be invaluable for understanding the mental health state of residents of Riyadh and inform the adoption of evidence-based policies, improving mental health literacy and developing an integrated care model within the primary care system to meet the needs of the target population and address the current treatment gap in Saudi Arabia.

## 2. Materials and Methods

### 2.1. Study Design, Setting, and Participant Selection

The cross-sectional study was done from the month of March to July 2023. A multi-stage cluster sampling was used to get a representative sample of people visiting primary healthcare centers (PHCs) in Riyadh. Firstly, the Riyadh region was divided into three health clusters. Health Cluster 2 was chosen because it has a diversified population and the healthcare system is highly developed (with an estimated population of 3.7 million individuals and 103 PHCs). The stratified random sampling technique was then used to select 48 PHCs among Health Cluster 2. This assisted in achieving a balanced representation of PHCs, both in the urban and the suburban areas. Finally, systematic random sampling was performed to choose the participants among 48 sampled PHCs. The multi-stage approach was meant to create a sample, which can provide a real representation of the whole population in terms of primary healthcare services within this specific region of Riyadh, to reduce the selection bias and maximize the generalizability of the findings.

### 2.2. Sample Population and Eligibility

The study sample comprised all people over the age of 18 who visited the selected PHCs in the city of Riyadh during the study period. This included Saudi and non-Saudi residents. The exclusion criteria were employed to maintain sample integrity. Patients under the age of 18 as well as the healthcare professionals or the employees of the PHCs were eliminated. In addition, the individuals that did not provide informed consent were also removed. The data collector approached visitors in the waiting room who met the inclusion criteria to fill out an electronic survey.

### 2.3. Questionnaire Development and Content

The study instrument has been developed in collaboration between the Central Health Services Reform Management Team and the consultants from the various regions of Saudi Arabia. This was done as part of a larger project to transform the health system by evaluating the health perceptions, behaviors, and also priorities of the people. The questionnaire (Supplementary Materials) was designed in a manner that incorporates a wide interest in factors influencing the health outcomes and healthcare utilization. It addressed self-reported health status, health priorities, and health behaviors like smoking, consumption of fast food, and exercise. In addition, the survey collected sociodemographic information, including age, education level, employment status, and family characteristics, as well as medical history variables such as hypertension, diabetes, obesity, hypercholesterolemia, and heart disease. Most questionnaire items were scored in a binary format (0 = No, 1 = Yes), including mental health status, comorbidities, insurance coverage, smoking, and other behavioral variables. Health status was assessed using a Likert scale ranging from “Poor” to “Excellent,” with higher scores indicating better perceived health. To assess mental health status, participants were asked whether they had ever been diagnosed with a mental health disorder by a healthcare professional. The question was phrased as: “Have you ever been diagnosed with a mental health disorder by a doctor or other healthcare professional?” with the response options of “Yes” or “No.” This item aimed to capture self-reported physician-diagnosed mental health conditions among participants. The questionnaire also included questions regarding insurance coverage and other health-related characteristics. Additionally, cognitive debriefing was conducted with a small sample of participants from the target population to ensure that all questions were clearly understood, culturally appropriate, and interpreted as intended. Feedback from this phase was used to refine the wording and structure of the questionnaire before pilot testing.

### 2.4. Reliability and Validity Testing

The questionnaire underwent rigorous evaluation for reliability and validity. A multidisciplinary panel of 15 experts, including psychologists, public health specialists, statisticians, and other relevant stakeholders, reviewed the content for clarity, relevance, and appropriateness. The Content Validity Index (CVI) for individual items ranged from 0.87 to 1.00, and the overall scale-level CVI (S-CVI) was 0.94, indicating excellent content validity. Additionally, face validity was assessed during pilot testing with 200 participants, who evaluated the clarity and understandability of the questions. The Face Validity Index (FVI) was calculated as 0.91, demonstrating that the items were well-understood and interpreted as intended. Questions that were unclear, difficult, or non-specific were revised based on the feedback from the focus groups to ensure clarity for participants of the main study. The test–retest reliability was examined by re-administering the survey to 100 respondents of the pilot study via telephone, yielding a reliability coefficient of 0.83, thereby confirming high consistency. To ensure linguistic accuracy, the questionnaire was translated to Arabic and subsequently back to English, maintaining the integrity and meaning across languages.

### 2.5. Initial Testing and Rationale for Location

The pilot study was carried out in Hail City, which was chosen by the Central Health Services Reform Management Team based on its demographics and health profile, which shows similarity to the rest of the Saudi population. This made it an ideal place to do preliminary testing. The pilot recruited 100 patients and 20 participants to study groups to measure the questionnaire clarity or difficulty. The unclear, difficult and non-specific questions were rewritten based on the feedback of the focus groups so that they could be easily understood by the participants of the main study.

After the revisions, the test–retest reliability was measured by re-administering the revised survey to the same 100 respondents, over the phone. The reliability coefficient was 0.83, which proved the strength of the questionnaire and its respectable face validity. It was done in January 2023, nearly a month prior to the commencement of the main data collection in Riyadh in March 2023. To ensure linguistic accuracy, the questionnaire was translated to Arabic and back to English, which was done carefully, making the tool ready to be used on large scale across Riyadh and other health clusters in Saudi Arabia.

### 2.6. Data Collection and Participant Information

The survey was done electronically in the presence of an interviewer. Data collectors used iPads or Android gadgets to administer the questionnaire to participants at primary healthcare clinics in Riyadh. The data collectors made sure that the participants were 18 years or older before asking them to take part. Eligibles were then contacted, and informed consent was obtained by clarifying the purpose of the study. The questionnaire was voluntary in all respects. The data collectors therefore provided the questionnaire to the consenting individuals. The survey collected vast information on sociodemographic variables (age, gender, family size, marital status, education, and employment), behavioral variables (smoking, fast foods, and physical activities) and the presence of comorbidities (hypertension, diabetes, and obesity). The final sample of this analysis consisted of 14,239 participants who completed the survey.

### 2.7. Statistical Analysis

The distribution of the data was assessed using histograms, and descriptive statistics were used to summarize the study variables. Continuous variables that were normally distributed, such as age, were presented as means and standard deviations. For the purpose of analysis, age was categorized into two groups: below 50 years and 50 years or above. This categorization was chosen to facilitate meaningful interpretation of mental health risk across age groups and to align with prior literature indicating differential patterns of mental health disorders in younger versus older adults. Categorical variables, including insurance coverage, health status, marital status, employment status, and educational level, were summarized using frequencies and proportions.

The primary outcome variable was the self-reported prior diagnosis of a mental health disorder (Yes/No). Potential explanatory variables were identified a priori based on existing literature on mental health determinants and theoretical relevance, including sociodemographic characteristics, behavioral factors, and comorbid health conditions. Logistic regression analyses were conducted to examine associations between predictors and mental health disorders. Univariable logistic regression models were first performed to assess the crude association between each independent variable and the outcome. These analyses were used as a complementary exploratory step rather than as the sole basis for variable selection. The variable selection strategy for the multivariable logistic regression was guided primarily by domain knowledge and literature review. Variables considered as potential confounders included sociodemographic factors (age, sex, education, marital status, employment, and insurance coverage) and key behavioral factors (smoking, diet, physical activity), while comorbid health conditions were treated as main predictors of interest. Additional variables with *p*-values < 0.25 in the univariable analysis were also considered for inclusion, as recommended in statistical literature for exploratory screening [20], to avoid excluding potentially relevant predictors. Subsequently, multivariable logistic regression models, including all relevant predictors identified a priori, were used to determine independent associations while adjusting for potential confounders. There were no missing observations for any variables included in the model; therefore, no imputation was required. The level of significance was set at *p* < 0.05, and results are presented as adjusted odds ratios (AORs) with 95% confidence intervals (CIs). All statistical analyses were performed using SPSS version 26.0 (IBM Corp., Armonk, NY, USA).

## 3. Results

### 3.1. Baseline Characteristics of Study Participants

Table 1 shows the sociodemographic and health characteristics of 14,239 Saudi residents who are included in the current mental health research. The average age of the respondents was 59.8 (SD:16.35) years, and a significant proportion (66%) of respondents were 50 years or above. Educational attainment was comparatively high with 68.3% of the respondents having a college education or higher. There were more females (56.6%) than males in the sample and the majority of people were married (65.3%). A little more than half (51.4%) of the respondents were employed. When it came to self-reported health status, a combined 69.3% indicated they had either excellent health (33.7%) or a very good health (35.6%). Nonetheless, a significant percentage (75.7%) experienced the lack of health insurance cover. The percentage of the respondents who participated in exercise was 60.7%. Among the comorbidities and behavioral factors, 27.7% of the participants were smokers, followed by diabetes (12.4%), hypertension (11.1%), and hypercholesterolemia (10.6%). It was found that 5.2% of the cohort had obesity, and 4.9% had heart disease. Table 1 indicates that 2.5% (95% CI: 2.24–2.76%) of respondents reported having previously been diagnosed with a mental health disorder by a healthcare professional.

### 3.2. Sociodemographic Determinants of Mental Health Disorders

Table 2 describes the sociodemographic factors related to mental health disorders among Saudi residents visiting primary healthcare centers in Riyadh, according to univariate and multivariable analyses (n = 14,239).

The factors which were significantly related to the mental health disorders (*p*-value of <0.25) in the univariate analysis included age, sex, education and insurance coverage. To be more precise, the odds of mental disorders were significantly higher among people aged under 50 years than among those aged 50 years or older (OR = 1.50, 95% CI: 1.21–1.85, *p* < 0.001). There were two-fold higher odds of having mental health disorders among females than males (OR = 2.00, 95% CI: 1.58–2.53, *p* < 0.001). Those who had at least college education were inclined towards greater odds of mental illnesses (OR = 1.23, 95% CI: 0.97–1.56, *p* = 0.08). Moreover, the coverage of health insurance was linked to almost twice the odds of mental health disorders (OR = 1.85, 95% CI: 1.49–2.30, *p* < 0.001). Univariate analysis did not show any significant associations with marital status and employment status (*p* = 0.40 and *p* = 0.96, respectively).

In the multivariable analysis (*p*-value cutoff of <0.05), age, sex, and insurance coverage were found to be significant independent determinants of mental health disorders. On adjustment of other factors, individuals under the age of 50 years still had much higher adjusted odds of having mental health disorders than individuals who are 50 years and above (AOR = 1.47, 95% CI: 1.19–1.83, *p* < 0.001). Women were always found to have higher adjusted odds of having mental health disorders than men (AOR = 1.98, 95% CI: 1.56–2.50, *p* < 0.001). On the same note, insurance coverage was also a strong predictor, with the insured experiencing a significantly greater adjusted odds of mental health disorders (AOR = 1.85, 95% CI: 1.48–2.30, *p* < 0.001). In the multivariable model, educational attainment was no longer a significant predictor (*p* = 0.14). The multivariable analysis did not include marital and employment status because they were found to be non-significant in the univariate analysis.

### 3.3. Behavioral Risk Factors and Comorbidities Associated with Mental Health Disorders

Table 3 presents the behavioral risk factors and comorbidities associated with mental health disorders among Saudi residents, based on univariate and multivariable analyses (n = 14,239).

In the univariate analysis, all investigated factors demonstrated a significant association with mental health disorders (*p* < 0.001). Smoking was strongly associated with mental health disorders, showing a nearly 7-fold increase in odds (OR = 7.09, 95% CI: 5.61–8.98). Fast food consumption also showed a significant association (OR = 1.76, 95% CI: 1.33–2.32). The presence of chronic conditions such as diabetes (OR = 5.21, 95% CI: 4.19–6.49), hypertension (OR = 13.76, 95% CI: 11.04–17.15), obesity (OR = 41.01, 95% CI: 32.53–51.70), hypercholesterolemia (OR = 23.86, 95% CI: 18.93–30.08), and heart disease (OR = 79.48, 95% CI: 62.13–101.68) were all highly associated with significantly elevated odds of mental health disorders.

For the multivariable analysis (with a *p*-value cutoff of <0.05), smoking, hypertension, obesity, hypercholesterolemia, and heart disease remained significant independent predictors of mental health disorders. After adjusting for other factors, smoking remained a significant risk factor (AOR = 4.45, 95% CI: 3.22–6.15, *p* < 0.001). Fast food consumption, however, was no longer a significant predictor in the multivariable model (AOR = 1.15, 95% CI: 0.78–1.71, *p* = 0.47). Diabetes also lost its significance in the multivariable analysis (AOR = 0.96, 95% CI: 0.66–1.39, *p* = 0.83). In contrast, hypertension (AOR = 2.32, 95% CI: 1.61–3.34, *p* < 0.001), obesity (AOR = 9.40, 95% CI: 6.96–12.70, *p* < 0.001), hypercholesterolemia (AOR = 2.84, 95% CI: 1.98–4.07, *p* < 0.001), and heart disease (AOR = 12.74, 95% CI: 9.25–17.56, *p* < 0.001) all continued to be highly significant predictors of mental health disorders (Table 3).

### 3.4. Model Fitness Results

To evaluate the adequacy and explanatory power of the multivariable logistic regression model, model fit statistics were examined. The final model demonstrated a −2 log likelihood value of 1582.600, indicating an improved model fit compared with the null model. The Cox & Snell R^2^ was 0.114, while the Nagelkerke R^2^ was 0.550, suggesting that the included predictors explained a moderate proportion of the variance in self-reported prior diagnosis of mental health disorders. Model estimation converged successfully after eight iterations with parameter estimates changing by less than 0.001, indicating stable model convergence.

## 4. Discussion

This study examined the prevalence of self-reported prior diagnosis of mental health disorders and their sociodemographic, behavioral, and comorbidity-related determinants among a large group of Saudi residents attending PHCs in Riyadh (n = 14,239). We found that 2.5% of participants reported having previously been diagnosed with a mental health disorder. The multivariable analysis revealed several important predictors, including younger age, female sex, health insurance coverage, smoking, hypertension, obesity, hypercholesterolemia, and heart disease.

The 2.5% prevalence observed in this study represents the proportion of participants reporting a prior diagnosis of a mental health disorder by a healthcare professional, rather than the true population prevalence of mental disorders. This distinction is important when comparing our findings with national and international estimates. Studies in the Middle East and North Africa (MENA) region have documented a substantial burden of mental illness [1,2]. Similarly, the Saudi National Mental Health Survey (SNMHS) reported that nearly one-third of Saudi residents experience a mental disorder at some point during their lifetime [21]. Another study among patients attending primary healthcare centers found a prevalence of 28.5% [22]. Additionally, overall, 24.7% and 35.9% of Saudi women experienced at least one mental disorder in the prior 12 months and at least once in their lifetime, respectively [23]. The lower estimate observed in our study should therefore be interpreted cautiously, as it reflects self-reported prior diagnoses rather than the actual prevalence of mental disorders in the population.

Several methodological factors may explain this discrepancy. First, the outcome variable in this study captures self-reported prior diagnosis of a mental health disorder, which inherently depends on prior contact with healthcare services and clinical recognition of the condition. Individuals who have experienced symptoms but have never sought professional care, have not received a formal diagnosis, or choose not to disclose their diagnosis may therefore be classified as not having a mental health disorder. Second, the reliance on self-report rather than standardized diagnostic instruments may lead to underestimation of the true burden, particularly in settings where stigma surrounding mental illness remains prevalent. Cultural beliefs, social stigma, and reluctance to disclose mental health conditions have been documented as barriers to accurate reporting in the Saudi context [11,24]. Third, the study population consisted exclusively of individuals attending primary healthcare centers, which may not fully represent the broader community, including individuals who seek care directly from specialized mental health facilities or those who do not engage with healthcare services at all. Taken together, these methodological considerations likely contribute to an underestimation of the true prevalence of mental disorders, and the findings should therefore be interpreted as reflecting the prevalence of self-reported prior mental health diagnoses among individuals attending primary healthcare centers, rather than the overall population prevalence of mental disorders.

Our multivariable analysis revealed age as an important determinant, where people that are younger than 50 years showed 1.47 times higher adjusted odds of mental health disorders than those aged 50 years or above. This observation is consistent with international trends that most mental health conditions often develop at a young age, with a substantial percentage of them presenting at early adulthood [25,26,27,28]. The pressures related to contemporary lifestyles, growing academic and occupation demands, social media exposure, excessive screen time, and shifting social patterns may have a disproportionate impact on younger age groups in Saudi Arabia [25,26]. On the other hand, older adults may have evolved coping strategies or may exhibit other mental health issue patterns that are less commonly manifested or diagnosed in primary care practices.

Female sex became a strong independent predictor, and females showed approximately two times higher adjusted odds of mental health disorders than males. This correlates well with the available global epidemiological data which regularly indicates higher prevalence rates of common mental disorders, especially anxiety and depression, among women [29,30,31]. It might be caused by a number of factors such as hormonal fluctuations, particular socioeconomic stressor, gender-specific cultural expectations, and the possibility of a higher tendency to seek help and report symptoms resulting in higher rates of diagnosis [32]. It is important to address these gender-specific vulnerabilities in order to establish specific mental health interventions.

Surprisingly, we discovered that the adjusted odds of having mental disorders were 1.85 times higher among people with health insurance coverage. This counter-intuitive result, in which insurance aligns with greater diagnosed prevalence, is probably due to greater access to healthcare services, including mental health assessments. Insured people tend to visit medical practitioners particularly during screening services [33,34], resulting in higher detection of mental health diseases that would otherwise go undetected in the uninsured group [35,36]. This underscores the importance of health insurance in the process of accessing care and revealing the actual burden of mental illness.

In addition to the sociodemographic factors, comorbidities and behavioral risk factors had significant roles. Smoking was a greatly independent predictor, with smokers’ adjusted odds of mental health disorders being 4.453 times higher. This association is well reported in the literature and indicates that the two-way relationship is complicated, with mental health conditions potentially predisposing individuals to smoking (e.g., self-medication) and, smoking, in turn, potentially increasing or causing the emergence of mental disorders due to neurochemical changes [37,38]. Fast food consumption and diabetes were significantly related in univariate analysis but lost their significance in the multivariable model, indicating that their impact on mental health could be mediated or confounded by other more influential factors.

More importantly, the existence of different chronic physical health conditions, hypertension, obesity, hypercholesterolemia, and heart disease, were all very significant independent predictors of mental health disorders in the multivariable analysis. These are extremely important findings highlighting the comorbidity between physical and mental health. The mechanisms that link these conditions are complex. Chronic physical conditions may be very distressing psychologically because of their symptoms, functional impairment, treatment burden, and existential issues, thus raising the risk of mental diseases, such as depression and anxiety [39,40,41,42,43,44,45,46]. On the other hand, mental disorders may adversely affect health behaviors (e.g., diet, exercise, medication adherence) and physiological systems (e.g., chronic stress, inflammation), and therefore make one susceptible to chronic physical diseases [47]. These bidirectional relationships emphasize the need for an integrated healthcare approach that incorporates routine mental health screening and support as part of chronic disease management within primary care settings.

Some comorbid conditions demonstrated unusually large odds ratios in the univariate analysis. However, these estimates were substantially attenuated after adjustment in the multivariable logistic regression model, indicating the presence of confounding by sociodemographic and clinical factors. For example, crude odds ratios for conditions such as heart disease and obesity decreased markedly after adjustment, suggesting that the initial large estimates reflected underlying differences in the distribution of covariates rather than independent effects. Additionally, sparse data in certain cross-tabulations and possible detection bias may have contributed to the inflated univariate estimates. These findings highlight the importance of multivariable adjustment when interpreting associations in observational cross-sectional studies.

### 4.1. Strengths and Limitations

The strengths of this study include a large sample size (n = 14,239) that was sampled at primary healthcare centers in Riyadh, which offers a strong dataset for the analysis of mental health determinants in a substantial portion of the Saudi Arabia. The independent predictors were identified by using both univariate and multivariable analyses that provided a deeper insight into relationships. Nevertheless, several limitations should be acknowledged. First, the cross-sectional design does not allow for causal inference, and therefore the observed associations cannot establish temporal or causal relationships between predictors and mental health disorders. As a result, reverse causation cannot be ruled out, meaning it is possible that the outcome may influence some predictors rather than vice versa. For example, some comorbidities or behavioral factors may represent consequences of mental health disorders or have bidirectional relationships, and adjusting for these variables could introduce overadjustment or collider bias. Analytical approaches such as causal inference methods and the use of directed acyclic graphs (DAGs) can help address potential reverse causation in future studies, but these were not applied in the current analysis. Second, the dependent variable was based on self-reported prior diagnosis of a mental health disorder, rather than a standardized clinical assessment or validated screening instruments such as structured diagnostic interviews or commonly used tools (e.g., PHQ-9 or GAD-7). This may introduce misclassification bias, as some individuals with undiagnosed mental health conditions may have been classified as not having a disorder, while others may have inaccurately reported prior diagnoses. Consequently, the prevalence of mental health disorders in this study may be underestimated. Additionally, self-reported health conditions and behavioral variables may be affected by recall bias and social desirability bias, particularly in cultural contexts where stigma surrounding mental illness may discourage disclosure. The study population consisted of individuals attending primary healthcare centers, which may also limit the generalizability of findings to individuals who do not utilize primary care services or who seek care directly from specialized mental health facilities. Finally, the absence of standardized diagnostic assessments may have influenced the precision of the prevalence estimates and the magnitude of observed associations. Future studies incorporating validated screening tools or clinical diagnostic interviews would help improve the accuracy of mental health disorder measurement in population-based research.

### 4.2. Policy Implications

The results of this research have profound policy implications on mental healthcare in Saudi Arabia. The close association between mental disorders and chronic physical ailments (hypertension, obesity, hypercholesterolemia, heart disease) require mental health to be incorporated into primary health care services. The routine screening of mental health in PHC facilities should be promoted in policies, especially for patients who come with chronic non-communicable illnesses. Educational sessions on mental health literacy and early recognition of mental health issues, as well as simple management of common mental disorders of primary care physicians and nurses are essential. Considering that the prevalence is higher in younger age groups and in females, specific public health campaigns on mental health for these groups are warranted. In addition, the relationship between health insurance and increased detection rates indicates that an increase in insurance coverage and increasing awareness about mental health benefits in insurance plans are an opportunity to promote access to care and decrease the unmet mental health service requirement. Lastly, combating the stigma that is rife in mental illness by educating the population and conducting awareness campaigns is crucial in order to promote help-seeking behaviors amongst the Saudi population.

### 4.3. Future Directions

Future studies ought to extend these results by using longitudinal study designs to assess the causal links between the specified predictors and mental health outcomes. This would facilitate the interpretation of mental illnesses’ trajectory and the development of comorbidities. The standardized diagnostic instruments may also be included in future research to obtain more precise prevalence estimates of the particular mental disorders. Another important area is the investigation of the effectiveness and cost-effectiveness of integrated mental health models in Saudi primary healthcare settings. There is also a need to conduct research on culturally sensitive interventions that can be used to overcome the special barriers to mental healthcare like stigma and cultural beliefs. Lastly, the analysis of the role of digital health solutions and telemedicine in enhancing access and outcomes of mental health, particularly among underserved populations, should be considered as a future research area.

## 5. Conclusions

The current study contributes to a better understanding of the prevalence and determinants of mental health disorders among attendees of primary healthcare in Riyadh. The results indicate the significance of timely detection and thorough treatment of mental conditions, especially among younger people, women, and individuals with chronic physical disorders. The prevalence of comorbidities is high, which requires an integrative approach to patient care that goes beyond the physical/mental health dichotomy. Longitudinal studies should also be incorporated in future research to determine causality, examine the particular subtypes of mental disorders, and examine the effectiveness of integrated mental health services in Saudi primary healthcare environments. The most important way to address the currently existing treatment gap and the societal stigma of mental illness is to help the Saudi population through public health campaigns to feel better in their mental health.

## Figures and Tables

**Table 1:** Sociodemographic characteristics of Saudi residents (n = 14,239).

Sociodemographic Factors		
Age, mean (SD)	59.8 (16.35)	
Categorical variables	n	%
Age groups		
<50 years	4848	34
At least 50 years	9391	66
Education		
Less than college education	4509	31.7
At least college education	9730	68.3
Sex		
Female	8062	56.6
Marital status		
Married	9300	65.3
Employment		
Employed	7317	51.4
Health status		
Excellent	4798	33.7
Very good	5076	35.6
Good	2815	19.8
Fair	1256	8.8
Poor	294	2.1
Insurance coverage		
Yes	3457	24.3
No	10,782	75.7
Exercise participation		
Yes	8641	60.7
No	5598	39.3
Behavioral factors and comorbidities		
Smoking status	3942	27.7
Obese	737	5.2
Diabetic	1765	12.4
Hypertensive	1580	11.1
Hypercholesterolemia	853	10.6
Heart disease	394	4.9
Mental health disorders	353	2.5

**Table 2:** Sociodemographic determinants of mental health disorders among Saudi residents visiting primary healthcare centers in Riyadh (n = 14,239).

Predictors	Univariate Analysis				Multivariable Analysis			
		95% CI		*p*-Value		95% CI		*p*-Value
	OR	LL	UL		AOR	LL	UL	
Age								
At least 50 years	1.00			<0.001	1.00			<0.001
<50 years	1.50	1.21	1.85		1.47	1.19	1.83	
Sex								
Male	1.00			<0.001				<0.001
Female	2.00	1.58	2.53		1.98	1.56	2.50	
Education								
Less than college education	1.00			0.087				0.141
At least college education	1.23	0.97	1.56		1.20	0.94	1.52	
Marital status								
Single	1.00			0.400	NA			
Married	1.10	0.88	1.38					
Employment status					NA			
Employed	1.00			0.966				
Unemployed	1.00	0.81	1.24					
Insurance coverage								
No	1			<0.001	1.00			<0.001
Yes	1.85	1.49	2.30		1.85	1.48	2.30	

OR: Odds ratio, LL: lower limit, UL: upper limit, NA: not applicable because the variable did not meet the threshold of *p*-value < 0.25 in the univariate analysis, AOR: adjusted odds ratios were calculated by mutually adjusting for all variables that were significant in univariate analysis.

**Table 3:** Behavioral risk factors and comorbidities associated with mental health disorders (n = 14,239).

	Univariate Analysis				Multivariable Analysis			
Predictors		95% CI		*p*-Value		95% CI		*p*-Value
	OR	LL	UL		AOR	LL	UL	
Smoking								
No	1.00			<0.001	1			<0.001
Yes	7.09	5.61	8.98		4.45	3.22	6.15	
Fast food consumption								
No	1.00			<0.001	1			0.468
Yes	1.76	1.33	2.32		1.15	0.78	1.71	
Diabetes								
No	1.00			<0.001	1			
Yes	5.21	4.19	6.49		0.96	0.66	1.39	0.834
Hypertension								
No	1.00			<0.001				<0.001
Yes	13.76	11.04	17.15		2.32	1.61	3.34	
Obesity								
No	1.00			<0.001	1			<0.001
Yes	41.01	32.53	51.70		9.40	6.96	12.70	
Hypercholesterolemia								
No	1.00			<0.001	1			<0.001
Yes	23.86	18.93	30.08		2.84	1.98	4.07	
Heart disease								
No	1.00			<0.001	1			<0.001
Yes	79.48	62.13	101.68		12.74	9.25	17.56	

AOR: Adjusted odds ratio, LL: lower limit, UL: upper limit. The model for multivariable analysis was mutually adjusted for other behavioral factors and comorbidities, age, and sex of the study participants.

## Data Availability

Data supporting this article is the property of King Fahad Medical City and cannot be available freely; however, we have added all related data within the article.

## Supplementary Materials

The following supporting information can be downloaded at: https://www.mdpi.com/article/10.3390/healthcare14060817/s1, Questionnaire: Health Need Assessment Survey of Primary HealthCare Centers visitors Within Riyadh Second Health Cluster.
