# Supplementary material for: The Prevalence and Comorbidities of Self-Reported Mental Health Disorders Among Primary Healthcare Attendees in Riyadh, Saudi Arabia: A Cross-Sectional Survey
Source: Healthcare (Basel). 2026 Mar 23;14(6):817. doi: 10.3390/healthcare14060817 (PMC13026869; doi:10.3390/healthcare14060817)
Supplement: Supplementary file 1 [file healthcare-14-00817-s001.zip › healthcare-4177428-supplementary.pdf]

Health Need Assessment Survey of Primary HealthCare Centers visitors Within Riyadh Second Health Cluster

استبيان لتقييم الاحتياجات الصحية لزائري المراكز الصحية التابعة للتجمع الصحي الثاني في منطقة الرياض

*Required

1. Data Collector Number *
رقم جامع/ة البيانات (رقم انجليزي)

Untitled Section

2. Primary Healthcare center *
اسم المركز الصحي

Mark only one oval.

- Alrawdah 2 الروضة ٢
- Alseddiq الصديق
- Alghadeer الغدير
- Alyasmeen الياسمين
- Alezdihar الإزدهار
- Alrabea الربيع
- Salahuddin صلاح الدين
- Almuruj المروج
- Almaseef المصيف
- Alyarmuk West اليرموك الغربي
- Al Munsiyah المونسية
- Qurtubah قرطبة
- Al_Nahhdah west النهضة الغربي
- Al_Nadheem north النظيم الشمالي
- Aljanadrya West الجنادرية الغربي
- As salam السلام
- As saadah السعادة
- An Nasim South النسيم الجنوبي
- Mashdhuba مشذوبة
- Barzan برزان
- Hewamdah حويمضة
- Al yamamh اليمامة
- Al-Qods القدس
- Al-Ghaat الغاط
- Albasira البصيرة
- Alfaihaa الفيحاء
- Al-Faisaliya الفيصلية
- Tameer تمير
- Al-Tuwaim التويم

- Oudat Sedair عودة سدير
- Rimah رماح
- Al-Ghilana الغيلانة

Demographic Characteristic

3. Participant Name *

اسم زائر المركز الصحي

____

4. Contact number *

(رقم التواصل(رقم انجليزي

____

5. Nationality *

الجنسية

Mark only one oval.

- Saudi
- Non Saudi

6. Neighborhood *

الحي

(باللغة الانجليزية

____

7. Gender *
الجنس

Mark only one oval.

- Female انثى
- Male ذكر

8. Marital Status *
الحالة الاجتماعية

Mark only one oval.

- Married متزوج/ة
- Single اعزب/عزباء

9. Age *
العمر (رقم انجليزي)

____

10. Highest level of Education Completed *
آخر شهادة تم حصولها

Mark only one oval.

- primary School الابتدائية
- High School الثانوية
- Did not finish High School لم يتم اكمال مرحلة الثانوية
- College Graduate بكالوريوس
- Master Graduate ماجستير
- Dectorate دكتوراه
- Diploma دبلوم
- Other آخرى

11. Number of people living in the household
عدد الاشخاص المقيمين في البيت

Mark only one oval.

- 1_2
- 3_5
- 6 or more

12. Number of adult aged 65 or older living in the household
عدد الاشخاص فوق ٦٥سنة الذين يعيشون في نفس المنزل

Mark only one oval.

- 0
- 1_2
- 3_5
- 6 or more

13. Number of children under 18 years living in the household
عدد الاطفال تحت ١٨سنة الذين يعيشون في نفس البيت

Mark only one oval.

- 0
- 1_2
- 3_5
- 6 or more

14. Employment Status *
الحالة الوظيفية

Mark only one oval.

- Employed full time موظف/ة دوام كامل
- Employed part time موظف/ة دوام جزئي
- Student طالب/ة
- Home Maker رب/ة منزل
- Retired متقاعد/ة
- Un employed less than a year غير موظف/ة لأقل من سنة
- Un employed more than a year غير موظف/ة لاكثر من سنة
- Un employed due to an illness or disability غير موظف/ة لمرض او اعاقة

15. Annual household income
مجموع الدخل السنوي لسكان المنزل

Mark only one oval.

- Less than 10,000SR
- 10,000 to19,999SR
- 20,000 to 29,999 SR
- 30,000 to 39,999 SR
- 40,000 to 49,999 SR
- 50,000 to 59,999 SR
- 60,000 to 69,999 SR
- 70,000 to 79,999 SR
- 80,000 to 89,999 SR
- 90,000 to 99,999 SR
- 100,000 to 149,999 SR
- 150,000 SR or more

Social Determinants of Health

16. Is Living place rent or own *
هل مكان المعيشة مستأجر أو مُلك

Mark only one oval.

- Own ملك
- Rent مستأجر
- Other آخرى

17. What sources do you use to obtain most health-related information? (check all that apply) *
ما هي المصادر التي تستخدمها للحصول على معظم المعلومات المتعلقة بالصحة؟ (يمكنك اختيار اكثر من اجابة)

Tick all that apply.

- [ ] Doctor/nurse/pharmacists الطبيب/الممرضة/الصيدلي
- [ ] Newspaper/magazine/TV الصحيفة/المجلات/التلفاز
- [ ] Ministry of Health وزارة الصحة
- [ ] Mosques المسجد
- [ ] School المدرسة
- [ ] Internet الانترنت
- [ ] Social Media منصة التواصل الإجتماعي
- [ ] Friends or family الأصدقاء أو العائلة
- [ ] Other آخرى

18. Check all of the following that describe your home or your household *
(تحقق مما يلي الذي يصف منزلك يمكن(يمكنك اختيار أكثر من اجابة

Tick all that apply.

- [ ] Smoke detector كاشف الدخان
- [ ] Carbon monoxide detector كاشف أول أكسيد الكربون
- [ ] Primary source of drinking and cooking water is a private well or Cistern المصدر الأساسي لمياه الشرب والطهي هو بئر خاص أو صهريج
- [ ] Food is put back into the refrigerator within the 2 hours after a meal يُعاد الطعام إلى الثلاجة في غضون ساعتين بعد الوجبة
- [ ] Trash removed at least weekly تتم إزالة المهملات أسبوعيًا على الأقل
- [ ] Septic tank خزان للصرف الصحي
- [ ] Pet (dog, cat, reptile) حيوان أليف (كلب ، قطة ، زواحف)
- [ ] Family fire safety plan /evacuation plan خطة سلامة الأسرة / خطة الإخلاء
- [ ] Children know how to dial 911 for emergencies يعرف الأطفال كيفية الاتصال برقم 911 في حالات الطوارئ
- [ ] Children know their phone number and address يعرف الأطفال رقم هاتفهم وعنوانهم
- [ ] Have one or more fire extinguishers وجود طفاية حريق واحدة أو أكثر
- [ ] Have one or more types of aerobic exercise equipment امتلك نوعًا واحدًا أو أكثر من معدات التمارين الهوائية
- [ ] Internet access خدمة الإنترنت
- [ ] Provide care for an older adult توفير الرعاية لكبار السن

19. choose how much of a problem you think each item listed below is for your community *
اختر مقدار المشكلة التي تعتقد أن كل عنصر مدرج أدناه يخص مجتمعك

Mark only one oval per row.

	Serious Problem مشكلة خطيرة	Moderate Problem مشكلة متوسطة	Not a Problem ليست مشكلة	Not Sure غير متأكد
**Alcohol /drug use تعاطي الكحول / المخدرات**	⬭	⬭	⬭	⬭
**Ambulance serices خدمات الإسعاف**	⬭	⬭	⬭	⬭
**Asthma /respiratory disorders / الربو اضطرابات الجهاز التنفسي**	⬭	⬭	⬭	⬭
**Eating disorders اضطرابات الاكل**	⬭	⬭	⬭	⬭
**Cancer السرطان**	⬭	⬭	⬭	⬭
**Child care/Day Care (safe, affordable, available) رعاية الأطفال / الرعاية النهارية (آمنة ، ميسورة التكلفة ، متوفرة)**	⬭	⬭	⬭	⬭
**Child abuse أساءةالأطفال**	⬭	⬭	⬭	⬭
**Clean water/water pollution المياه النظيفة / تلوث المياه**	⬭	⬭	⬭	⬭
**Crime الجريمة**	⬭	⬭	⬭	⬭

**Domestic violence العنف المنزلي**				
**School systems أنظمة المدرسة**				
**Elder abuse إساءة معاملة المسنين**				
**Elder day care (safe, affordable, available) رعاية المسنين النهارية (آمنة ، وبأسعار معقولة ، ومتاحة)**				
**Firearms الأسلحة النارية**				
**Healthcare affordability القدرة على تحمل تكاليف الرعاية الصحية**				
**Health care availability توافر الرعاية الصحية**				
**Heart disease امراض القلب**				
**High blood pressure ارتفاع ضغط الدم**				
**Strokes السكتة الدماغية**				
**Highway safety سلامة الطرق السريعة**				
**HIV/AIDS فيروس نقص المناعة البشرية**				

الإيدز /				
**Infant health** صحة الرضع	◯	◯	◯	◯
**Infectious disease** امراض معدية	◯	◯	◯	◯
**Job availability** توفر وظائف	◯	◯	◯	◯
**Job security** الآمان الوظيفي	◯	◯	◯	◯
**Mental illness** الامراض العقلية	◯	◯	◯	◯
**Nursing home care (safe, affordable, available)** رعاية التمريض في المنزل (آمنة ، وبأسعار معقولة ، ومتاحة)	◯	◯	◯	◯
**Overweight adults** البالغين الذين يعانون من زيادة الوزن	◯	◯	◯	◯
**Overweight children** الأطفال يعانون من زيادة الوزن	◯	◯	◯	◯
**Prenatal health** صحة ما قبل الولادة	◯	◯	◯	◯
**Recreation opportunities** فرص ترفيهية	◯	◯	◯	◯
**Secondhand smoke** التدخين السلبي	◯	◯	◯	◯
**School**	◯	◯	◯	◯

**violence عنف المدرسة**				
**Services for disabled خدمات للمعاقين**	◯	◯	◯	◯
**Sewage treatment معالجة مياه الصرف الصحي**	◯	◯	◯	◯
**Sexually transmitted infections الأمراض المتقولة جنسيا**	◯	◯	◯	◯
**Smokeless tobacco التبغ**	◯	◯	◯	◯
**Smoking التدخين**	◯	◯	◯	◯
**Suicide الانتحار**	◯	◯	◯	◯
**Trash /solid waste management / إدارة النفايات النفايات الصلبة**	◯	◯	◯	◯
**Unemployment العطالة**	◯	◯	◯	◯

Perceived General Health

20. Your current health status *
كيف تصف حالتك الصحية

Mark only one oval.

- Poor ضعيفة
- Fair مقبولة
- Good جيدة
- Very Good جيد جدا
- Excellent ممتازة

21. How many days have YOU been too sick to work or carry out your usual activities during the past 30 days *
كم عدد الأيام التي كنت مريضًا فيها لدرجة تمنعك من العمل أو القيام بأنشطتك المعتادة خلال الثلاثين يومًا الماضية

Mark only one oval.

- None لم أمرض
- 1-2 days يوم إلى يومان
- 3-5 days ثلاثة إلى خمسة أيام
- 6-10 days ستة إلى عشرة أيام
- More than 10 days أكثر من عشرة أيام

22. Have YOU ever had health issues due to any of the following? (check all that apply) *
هل عانيت من قبل من مشاكل صحية بسبب أي مما يلي؟ (يمكنك اختيار أكثر من إجابة)

Tick all that apply.

- [ ] Alcohol abuse الكحول
- [ ] Lack of pregnancy care عدم وجود رعاية حمل
- [ ] Stress القلق
- [ ] Drug abuse/addiction اساءة استخدام الادوية أو الادمان عليها
- [ ] Family violence العنف الأسري
- [ ] None of the above لم أتعرض إلى مشكلة صحية بسس هذه الخيارات

Insurance Information

23. Participant Insurance coverage and/or family *
هل يوجد تأمين طبي لك و/أو لأفراد العائلة

Mark only one oval.

- No insurance for me and my family لايوجد تأمين طبي لي أو لعائلتي *Skip to question 26*
- I have insurance for me and/or my family يوجد تأمين طبي لي و/أو لأفراد العائلة

24. If You and/or your family have insurance, what kind of Insurance
في حال وجود تأمين، ماهو تصنيف التأمين الطبي لك و/أو لأفراد عائلتك

Mark only one oval.

- Health صحي
- Dental اسنان
- Vision نظر
- Not applicable لاينطبق

25. If you and/or family have insurance, what is the coverage type
في حال وجود تأمين، ماهو نوع التأمين الطبي لك و/أو لأفراد عائلتك

Mark only one oval.

- Self Insured تأمين ذاتي
- Employer-provided مقدم من صاحب العمل
- Other أخرى
- Don't know لا أعلم
- Not applicable لاينطبق

HealthCare Utilization

26. Where do YOU go for routine health care? (check all that apply) *
(اين تذهب روتينياً لتلقي رعاية الصحية (يمكنك اختيار اكثر من اجابة

Tick all that apply.

- [ ] Hospital Emergency Room غرفة الطوارئ في المستشفى
- [ ] Urgent Care Center مركز الرعاية العاجلة
- [ ] Chiropractor مقوم العظام
- [ ] PHC مركز صحي
- [ ] Eye doctor طبيب العيون
- [ ] Dentist طبيب الأسنان
- [ ] Others أخرى
- [ ] Do not seek health care لا أتلقى رعاية طبية

27. If you have children in household, where do the children currently living in your home go for routine health care? (check all that apply)
(إذا لديك أطفال بالمنزل، اين يذهب الاطفال روتينياً لتلقي رعاية الصحية (تحقق من كل ما ينطبق

Tick all that apply.

- [ ] Hospital Emergency Room غرفة الطوارئ في المستشفى
- [ ] Urgent Care Center مركز الرعاية العاجلة
- [ ] Chiropractor مقوم العظام
- [ ] PHC مركز صحي
- [ ] Eye doctor طبيب العيون
- [ ] Dentist طبيب الأسنان
- [ ] Others أخرى

28. How often you seek medical care in private sector? *
كم مرة تسعى للحصول على رعاية طبية في القطاع الخاص؟

Mark only one oval.

- ( ) Always دائماً
- ( ) Sometimes أحياناً
- ( ) Seldom نادراً
- ( ) Never لا أذهب إلى القطاع الخاص لتلقي الرعاية الطبية

29. If you seek medical care in the private sector, why?
إذا كنت تبحث عن رعاية طبية في القطاع الخاص فلماذا؟

Mark only one oval.

- Services not available in public sector الخدمات غير متوفرة في القطاع العام
- Better quality جودة أفضل
- I have insurance لوجود تأمين
- Closer to work/home أقرب إلى مكان عملي/منزلي
- Too hard to get appointment with public doctors صعوبة الحصول على موعد مع طبيب في القطاع العام
- Do not apply لا ينطبق

30. How often do you travel outside of your Region for medical care? *
كم مرة تسافر خارج منطقتك لتلقي الرعاية الطبية؟

Mark only one oval.

- Always دائماً
- Sometimes أحياناً
- Seldom نادراً
- Never لا أسافر خارج منطقتي لتلقي الرعاية الطبية

31. If you travel outside of your county for medical care, why? *
إذا كنت تسافر خارج دولتك للحصول على رعاية طبية ، فلماذا؟

Mark only one oval.

- Services not available in my county الخدمات غير متوفرة في بلدي
- Better quality elsewhere جودة أفضل في مكان آخر
- Recently moved to this county انتقلت مؤخرا إلى هذه الدولة
- Too hard to get appointment with local doctor من الصعب جدا الحصول على موعد مع طبيب محلي
- Other اخرى
- N/A (do not travel outside of my county for medical care) (لا أسافر خارج مقاطعتى للحصول على رعاية طبية)

32. If you travel outside of your Region for medical care, what services do you seek? (check all that apply)
إذا كنت تسافر خارج منطقتك للحصول على رعاية طبية ، فما هي الخدمات التي تبحث عنها؟ (يمكنك اختيار اكثر من اجابة)

Tick all that apply.

- [ ] Medical/doctor appointments للمواعيد الطبية
- [ ] Outpatient treatment للعلاج في العيادات الخارجية
- [ ] Hospitalization العلاج في المستشفيات
- [ ] Dental appointments لمواعيد طب الأسنان
- [ ] Laboratory order tests لعمل تحاليل مختبر
- [ ] X-rays أشعة
- [ ] Vision appointments لمواعيد فحص النظر
- [ ] Do not apply لاينطبق
- [ ] Other: ____________________

Accessibility of Healthcare Services

33. How often are YOU able to visit a doctor when needed? *
هل لديك القدرة على زيارة الطبيب عند الحاجة

Mark only one oval.

- Always دائماً
- Sometimes أحياناً
- Seldom نادراً
- Never أبداً

34. If you answered seldom or never , choose why were not always able to visit a doctor when needed:
إذا كانت إجابتك نادرًا أو أبدًا ، فاختر سبب عدم قدرتك دائمًا على زيارة الطبيب عند الحاجة

Mark only one oval.

- Couldn't get appointment لم أستطع حجز موعد
- Lack of transportation وسائل النقل
- Doctors is too far away بعد المسافة
- Other اخرى
- Not Applicable لاينطبق

Disease Prevention Measures

35. Last routine doctors visit *
آخر زيارة روتينية للطبيب

Mark only one oval.

- Within last 12 months خلال ١٢ شهر الماضية
- Within last 13_18 months خلال ١٣-١٨ شهر الماضية
- Within last 19_24 months خلال ١٩_٢٤ شهر الماضية
- Between 2 and 5 years بين سنتين إلى ٥ سنوات
- More than 5 years أكثر من خمس سنوات
- Never had a routine visit لم يكن لدي زيارة روتينية

36. When was YOUR last routine dentist visit to get a checkup (not for an emergency)? *
أخر زيارة لك روتينية لطبيب الأسنان (لاتحتسب الحالة الطارئة)

Mark only one oval.

- Within the last year خلال السنة الماضية
- 1_2 years ago خلال سنة إلى السنتين الماضية
- 3_5 years ago خلال ٣_٥ سنوات الماضية
- More than 5 years أكثر من خمس سنوات
- Never had a routine dental visit لم يكن لدي زيارة روتينية لطبيب الأسنان

37. Select any of the following preventive procedures YOU have had in the last year (check all that apply) *
(اختار أي من الإجراءات الوقائية التي اجريتها السنة الماضية (يمكن اختيار أكثر من اجراء

Tick all that apply.

- [ ] Mammogram الماموجرام
- [ ] Pap smear مسحة عنق الرحم
- [ ] Glaucoma test اختبار الجلوكوما
- [ ] Flu shot تطعيمة الإنفلونزا
- [ ] Colon/rectal examination فحص القولون / المستقيم
- [ ] Blood pressure check قياس ضغط الدم
- [ ] Blood sugar check قياس سكر الدم
- [ ] Skin cancer screening فحص سرطان الجلد
- [ ] Prostate cancer digital screen فحص بالشاشة رقمية لسرطان البروستاتا
- [ ] Prostate cancer PSA blood screen فحص سرطان البروستاتا المستضد البروستاتي النوعي
- [ ] Cholesterol screen فحص الكوليسترول
- [ ] STI (sexually Transmitted Infections) screening فحص العدوى المنقولة جنسيًا
- [ ] Vision screening فحص النظر
- [ ] Hearing screening فحص السمع
- [ ] Cardiovascular screening فحص القلب والأوعية الدموية
- [ ] Bone density test اختبار كثافة العظام
- [ ] Dental exam كشف الاسنان
- [ ] None لم اعمل اجراء وقائي خلال السنة الماضية

38. When/If you were ever pregnant, did you receive prenatal care?
اذا سبق لكِ الحمل، هل استخدمتي رعاية ماقبل الولادة

Mark only one oval.

- ( ) Yes نعم
- ( ) No لا
- ( ) Not sure غير متأكدة
- ( ) Does not apply لاينطبق *Skip to question 33*

39. When/If you ever gave birth, did you breastfeed?
هل سبق لكِ الارضاع الطبيعي بعد الولادة

Mark only one oval.

- Yes نعم
- No لا
- Not sure غير متأكدة
- Does not apply لاينطبق

40. If you have children in household, Are the children currently living in your home current on their immunizations?
إذا كان لديك أطفال بالمنزل ، فهل الأطفال الذين يعيشون في منزلك حاليًا يتلقون التطعيمات؟

Mark only one oval.

- yes نعم
- No لا
- Do not know لا أعلم
- Does not apply لاينطبق *Skip to question 43*

Chronic Diseases

41. Have YOU been diagnosed by a doctor with any of the following health problems or disease? *

هل تم تشخيصك من قبل طبيب بأي من المشاكل الصحية أو الأمراض التالية؟

Tick all that apply.

	No لا	Yes نعم	I see a doctor اتابع مع دكتور	I am taking medications or getting treatment أنا أتناول الأدوية أو أتلقى العلاج	I feel the disease is well managed أشعر بأن المرض مُدار بشكل جيد
**Diabetes داء السكري**	☐	☐	☐	☐	☐
**Stroke السكتة الدماغية**	☐	☐	☐	☐	☐
**High blood pressure ضغط دم مرتفع**	☐	☐	☐	☐	☐
**High cholesterol ارتفاع الكوليسترول**	☐	☐	☐	☐	☐
**Heart disease مرض قلبي**	☐	☐	☐	☐	☐
**Cancer سرطان**	☐	☐	☐	☐	☐
**Asthma الربو**	☐	☐	☐	☐	☐
**Respiratory تنفسي**	☐	☐	☐	☐	☐
**lung disease امراض الرئة**	☐	☐	☐	☐	☐
**Kidney disease امراض الكلى**	☐	☐	☐	☐	☐
**Obesity السمنة**	☐	☐	☐	☐	☐
**Liver**	☐	☐	☐	☐	☐

**disease** امراض الكبد					
**Arthritis** التهاب المفاصل	☐	☐	☐	☐	☐
**Migraine headaches** صداع تصفي	☐	☐	☐	☐	☐
**Mental disorders** أمراض عقلية	☐	☐	☐	☐	☐
**Hepatitis** التهاب الكبد	☐	☐	☐	☐	☐
**Tuberculosis** مرض الدرن	☐	☐	☐	☐	☐
**Epilepsy** الصرع	☐	☐	☐	☐	☐
**Lupus** الذئبة	☐	☐	☐	☐	☐
**Sickle cell anemia** فقر الدم المنجلي	☐	☐	☐	☐	☐
**Glaucoma** المياه الزرقاء في العين	☐	☐	☐	☐	☐
**Gonorrhea** السيلان	☐	☐	☐	☐	☐
**HIV/AIDS** فيروس نقص المناعة البشرية / الإيدز	☐	☐	☐	☐	☐
**Dental health problems** مشاكل صحة الأسنان	☐	☐	☐	☐	☐
**Hearing**	☐	☐	☐	☐	☐

**disorders اضطرابات السمع**					
**Eye disorders اضطرابات العين**	☐	☐	☐	☐	☐
**Memory loss فقدان الذاكرة**	☐	☐	☐	☐	☐
**Sinus problems مشاكل الجيوب الأنفية**	☐	☐	☐	☐	☐

42. Check all the special needs children in your home have faced (Check all that apply)

اذا لديك أطفال في المنزل، حدد كل ماينطبق من الاحتياجات الخاصة الاتية التي يواجهها الطفل(يمكنك اختيار أكثر من اجابة)

Tick all that apply.

- ☐ My children (child living in my hose) do not have any special needs أطفالي (الطفل الذي يعيش في المنزل) ليس لديهم أي احتياجات خاصة
- ☐ Attention deficit / hyperactivity disorder (AD/HD) اضطراب نقص الانتباه مع فرط النشاط (AD / HD)
- ☐ Autism توحد
- ☐ Blindness/ visual impairment العمى / ضعف البصر
- ☐ Cerebral palsy الشلل الدماغي
- ☐ Child who uses a wheelchair الطفل يستخدم كرسي متحرك
- ☐ Deaf / hearing loss الصمم / فقدان السمع
- ☐ Developmental delay تأخر النمو
- ☐ Down syndrome متلازمة داون
- ☐ Emotional disturbance الاضطراب العاطفي
- ☐ Epilepsy / Seizure disorder اضطراب الصرع / النوبات
- ☐ Intellectual disability (formerly mental retardation) (الإعاقة الذهنية (التخلف العقلي سابقاً)
- ☐ Learning disabilities صعوبات التعلم
- ☐ Speech and language impairment صعوبات التعلم
- ☐ Spina bifida السنسنة المشقوقة (انشقاق العمود الفقري)
- ☐ Traumatic brain injury إصابات في الدماغ
- ☐ Other: ____________________

Behavioral and Lifestyle Factors

43. In the following section, how often do YOU do the following? (*N/A= does not apply to you) *
في القسم التالي ، كم مرة تفعل ما يلي؟
(* N / A = لا ينطبق عليك)

Mark only one oval per row.

	Almost Always دائماً	Sometimes أحياناً	Never أبداً	N/A* لاينطبق
**Wear a seat belt اربط حزام الامان**	⬭	⬭	⬭	⬭
**Wear a helmet when riding a motorcycle, scooter ارتداء خوذة عند ركوب دراجة نارية ، سكوتر**	⬭	⬭	⬭	⬭
**Drive the posted speed limit قيادة الحد الأقصى للسرعة المحددة**	⬭	⬭	⬭	⬭
**Eat at least 5 servings of fruits and vegetables each day تناول ما لا يقل عن 5 حصص من الفاكهة والخضروات كل يوم**	⬭	⬭	⬭	⬭
**Eat fast food more than once a week تناول الوجبات السريعة أكثر من مرة في الأسبوع**	⬭	⬭	⬭	⬭
**Exercise at a moderate pace at least 13 minutes per day 5 days per week تمرن بوتيرة معتدلة على الأقل 13 دقيقة يوميًا 5 أيام في الأسبوع**	⬭	⬭	⬭	⬭
**Smoking cigarettes تدخين السجائر**	⬭	⬭	⬭	⬭
**Chew tobacco مضغ التبغ**	⬭	⬭	⬭	⬭

**Are exposed to secondhand smoke in your home or work** تتعرض للتدخين السلبي في منزلك أو عملك	◯	◯	◯	◯
**Use illegal drugs** استخدم العقاقير المحظورة	◯	◯	◯	◯
**Wash hands with soap and water after using the restroom** غسل يديك بالماء والصابون بعد استخدام دورة المياه	◯	◯	◯	◯
**Wash hands with soap and water before preparing or eating meals** غسل يديك بالماء والصابون قبل تحضير أو تناول الوجبات	◯	◯	◯	◯
**Apply sunscreen before plan time outside** وضع الكريم الواقي من الشمس قبل التوجه للخارج	◯	◯	◯	◯
**Get a flu shot each year** احصل على لقاح الأنفلونزا كل عام	◯	◯	◯	◯
**Take vitamins pills or supplements daily** أتناول أقراص الفيتامينات أو المكملات الغذائية يوميًا	◯	◯	◯	◯
**Get enough sleep each night 7 to 9 hours** احصل على قسط كافٍ من النوم كل ليلة من 7 إلى 9 ساعات	◯	◯	◯	◯
**Feel stressed out** تشعر بالتوتر	◯	◯	◯	◯
**Feel happy about your life** تشعر بالسعادة في حياتك	◯	◯	◯	◯

**Feel lonely تشعر بالوحدة**				
**Worry about losing a job تقلق بشأن فقدان الوظيفة**				
**Feel safe in your community تشعر بالأمان في مجتمعك**				

44. Hight (CM)
الطول
الرقم انجليزي

45. Wight (KG)
الوزن
الرقم انجليزي

46. File Number

This content is neither created nor endorsed by Google.

Google Forms
